# The Masquelet induced membrane technique with PRP-FG-nHA/PA66 scaffold can heal a rat large femoral bone defect

**DOI:** 10.1186/s12891-024-07567-y

**Published:** 2024-06-08

**Authors:** Xiaoyu Wang, Yong Huang, Daqian Liu, Teng Zeng, Jingzhe Wang, Md Junaed Al Hasan, Wei Liu, Dawei Wang

**Affiliations:** 1 Department of Orthopedic Surgery, the First Affiliated Hospital of Harbin Medical University, 23 Youzheng Street, Nangang District, Harbin, 150001 Heilongjiang Province China; 2 Department of Orthopedic Surgery, the Affiliated Hospital of Qinghai University, Xining, Qinghai China; 3 Department of Orthopedic Surgery, the Second Affiliated Hospital of Harbin Medical University, Harbin, Heilongjiang China

**Keywords:** Masquelet membrane induction technology, platelet rich plasma, large bone defect, angiogenesis, bone regeneration

## Abstract

**Background:**

Masquelet membrane induction technology is one of the treatment strategies for large bone defect (LBD). However, the angiogenesis ability of induced membrane decreases with time and autologous bone grafting is associated with donor site morbidity. This study investigates if the PRP-FG-nHA/PA66 scaffold can be used as a spacer instead of PMMA to improve the angiogenesis ability of induced membrane and reduce the amount of autologous bone graft.

**Methods:**

Platelet rich plasma (PRP) was prepared and PRP-FG-nHA/PA66 scaffold was synthesized and observed. The sustained release of VEGFA and porosity of the scaffold were analyzed. We established a femur LBD model in male SD rats. 55 rats were randomly divided into four groups depending on the spacer filled in the defect area. “Defect only” group (*n* = 10), “PMMA” group (*n* = 15), “PRP-nHA/PA66” group (*n* = 15) and “PRP-FG-nHA/PA66” group (*n* = 15 ). At 6 weeks, the spacers were removed and the defects were grafted. The induced membrane and bone were collected and stained. The bone formation was detected by micro-CT and the callus union was scored on a three point system.

**Results:**

The PRP-FG-nHA/PA66 scaffold was porosity and could maintain a high concentration of VEGFA after 30 days of preparation. The induced membrane in PRP-FG-nHA/PA66 group was thinner than PMMA, but the vessel density was higher.The weight of autogenous bone grafted in PRP-FG-nHA/PA66 group was significantly smaller than that of PMMA group. In PRP-FG-nHA/PA66 group, the bone defect was morphologically repaired.

**Conclusion:**

The study showed that PRP-FG-nHA/PA66 scaffold can significantly reduce the amount of autologous bone graft, and can achieve similar bone defect repair effect as PMMA. Our findings provide some reference and theoretical support for the treatment of large segmental bone defects in humans.

## Introduction

The repair and reconstruction of large bone defect (LBD) is one of the most challenging problems for orthopedic surgeons. LBD is often caused by severe trauma, debridement after infection, bone tumor resection, and other reasons [1]. The treatment of LBD is difficult, especially when combined with infection and poor soft tissue conditions. Blood vessels have many biological functions such as nutrient supply mechanism, molecular signaling transmission and stem cell transport. Angiogenesis is crucial for bone regeneration. Lack of angiogenesis is one of the reason of hindering bone development, regeneration and proper systemic functioning [2]. Studies have shown that the reason why many bone defects are difficult to heal is precisely because of the difficulty of local blood vessel formation [3]. Therefore, the treatment of bone defect requires not only filling the defect, but also recovering the local angiogenesis ability.

In recent years, Masquelet membrane induction technology has become one of the treatment strategies for LBD due to its ability to promote the blood vessel formation of induced membrane [4]. The Masquelet membrane induction technology was proposed by Masquelet et al. in 2000. This technique consists of two stages of surgery: First, after complete debridement, the defect is filled with polymethyl methacrylate (PMMA) cement as a spacer. Second, after 6–8 weeks of implantation, the PMMA spacers were removed and followed by autologous bone grafted. At present, this technology has achieved good results in the treatment of large bone defects [5]. However, the technology has many drawbacks. First, as a traditional membrane induction medium, PMMA cement has little bone induction activity. Second, PMMA has thermogenic effect during the solidification process, which is easy to cause thermal damage to the surrounding tissues [6]. Third, the angiogenesis ability of the membrane induced by PMMA gradually decreases after implantation [7]. Last, after the removement of PMMA, the space left always needs large amount of autologous bone which will cause donor site morbidity and secondary deformity. Therefore, it is important to improve the angiogenesis ability of induced membrane and reduce the amount of autograft bone grafted in the second stage.

Platelet rich plasma (PRP), an autologous concentration of platelets from autologous whole blood by secondary centrifugation, has rapidly evolved as a focal point of interest in the realm of orthopedic therapeutic [8]. The autologous derivation of PRP offers both economic advantages and obviates potential immunogenic complications commonly linked to exogenous recombinant growth factors. Such benefits not only reduce the risk of disease transmission but also facilitate its application in surgery [9]. Upon activation, PRP will release an array of growth factors and cytokines, such as VEGFA, PDGF and TGF-β1, holding pivotal roles in processes like fracture healing, tissue reconstruction, and angiogenesis [10]. Many studies have shown that PRP can accelerate the healing process of a variety of refractory ischemic wounds such as chronic bed sore wounds and ulcer wounds [11, 12], PRP can also improve bone ischemic states such as femoral head ischemic necrosis and diabetic bone delayed union [13, 14].

Fibrinogen (FG) can form a membrane-like fibrous network structure after polymerization, which is often used to promote wound repair [15]. Due to its good biocompatibility, it is also commonly used as a delivery vehicle for growth factors. With the fibrin gradually degraded by fibrinolysis in vivo, the growth factors will be stably released, thus achieving a sustained effect. Fibrinogen has achieved good therapeutic effects in the treatment of lesions in bone or cartilaginous tissue [16]. Therefore, in this study, we plan to use fibrinogen to promote the sustained release of PRP related growth factors, thus maximizing the bioactivity of PRP.

In our study, a novel PRP-FG-nHA/PA66 scaffold was designed as an alternative to PMMA cement in Masquelet technology. Our study demonstrated the PRP-FG-nHA/PA66 scaffold could not only promote the angiogenesis of induced membrane, but also reduce the amount of autograft implanted in the second stage. Our study improved the Masquelet technology and provide some reference and theoretical support for the treatment of large segmental bone defects in clinical.

## Materials and methods

### Experimental animals

Male Sprague Dawley (SD) rats in this study were ordered from the Laboratory Animal Center of the Second Affiliated Hospital of Harbin Medical University (Harbin, Heilongjiang, China). All procedures were performed under the approval of the Animal Care and Use Committee of Harbin Medical University (IACUC NO.: 2,022,061). Rat were housed in groups of four and gave five days to acclimate to the housing facility. The temperature was 22 ± 2 ℃, humidity was 55%, and natural light-dark cycle. Animals were housed in 600 × 380 × 200 mm^3^ cages and rats were given maintenance food and water. The health status of the animals was monitored twice daily. All sections of this report adhere to the ARRIVE Guidelines 2.0 for reporting animal research.

### PRP preparation

PRP donor animals (SD rat, *n* = 40) were anesthetized with intraperitoneal injection of phenobarbital sodium (40 mg/kg) and then humanely euthanized. 5 animals as one experimental unit. The PRP was prepared by the two-step centrifugation method as previously described [17]. Briefly, the whole blood was drawn from rats by an intra-cardiac puncture, then the blood was anticoagulated and centrifuged for 15 min at 800 rpm at 20 °C. The upper plasma was collected and centrifuged again for 15 min at 2000 rpm at 20 °C. The upper layer was removed, leaving the PRP and buffy coat. The whole blood and PRP were taken for platelet counting to ensure that the number of platelets in PRP was 3–4 times higher than that in the whole blood.

### Scaffold preparation and lyophilization

The nHA-PA66 scaffold was synthesized as previously described [18]. Briefly, nHA-PA66 powders were mixed with ethanol at room temperature, the ratio of composite to ethanol (w: V) was varied from 0.6 to 0.8. Then the mixture was cast into the Teflon® mould and moved into oven at 80 ℃ for 3 days. During this period, phase inversion of ethanol was thermally induced. After ethanol gradually evaporated from the mould, the material became solidification. The porous scaffold was finally obtained after being dried at 100℃. The scaffold was cut into small pieces (5 × 5 × 5 mm^3^) with a 1 mm diameter groove in the middle and sterilized using ethylene oxide gas.

Fibrinogen solution was prepared by dissolving fibrinogen (Harbin Hanbang Medical Technology Co., Ltd. Harbin, China) in sterile water. The equal volume of PRP was mixed with the fibrinogen solution. The nHA/PA66 was fully immersed in the mixture for 5 min. Then calcium chloride (40 mmol/L) and thrombin (25 U/mL) were added to the mixture as activators. The reaction lasted for 30 min, and then the uncondensed liquid was removed.

The process of lyophilization was as follows:

Pre-cooling at -4 ℃ for 48 h;

Frozen at -80℃ for 24 h;

-70 ℃, 6.67 × 10^− 4^ kPa, 6 h;

After lyophilization, the samples were sterilized with ethylene oxide and stored at -20 ℃. Controls were established without fibrinogen and lyophilization.

### Sustained release of VEGFA

The scaffold was infiltrated in PBS at 37℃ and shaken thoroughly. The liquid was changed every 3 days and stored at -80℃. The concentration of VEGFA in the collected liquid was detected by ELISA according to the manufacturer’s instructions (Shanghai Enzyme-linked Biotechnology Co., Ltd. Shanghai, China).

### Scanning electron microscopy (SEM) observation

The samples were immobilized with 2.5% (v/v) glutaraldehyde solution at 4℃ for 4 h, dehydrated with a graded series of ethanol (30, 50, 70, 90, and 100%), freeze-dried for 48 h. The samples were finally coated with gold. The morphology of the scaffold were observed by scanning electron microscopy (HITACHI, Japan). The protocols were the same as our previous study [17].

### Porosity measurement

The porosity of the PRP-FG-nHA/PA66 scaffold was measured by liquid displacement method as previously described [19]. Ethanol was chosen as the displacing liquid because it easily penetrated into the pores of the scaffold without destroying the scaffold. The scaffold was completely immersed in a known volume (V1) of ethanol. The total volume of ethanol and ethanol - saturated scaffold was recorded as V2. The saturated scaffold was then removed and the volume of residual ethanol was recorded as V3. The volume of the saturated scaffold was V2–V3 and the volume of ethanol within the scaffold was V1–V3. The porosity of the scaffold was calculated as follows.

$$Porosity{\text{ }}\left( \% \right){\text{ }} = {\text{ }}\left( {V1{\text{ }} - {\text{ }}V3} \right){\text{ }}/{\text{ }}\left( {V2{\text{ }} - {\text{ }}V3} \right){\text{ }} \times {\text{ }}100$$

The experiments were performed 5 times and the average porosity value was obtained.

### Experiments on animals

Segmental bone defect was created in the middle of the right femur as previously described [20]. The rats were anesthetized with intraperitoneal injection of phenobarbital sodium (40 mg/kg). The individual rat was considered the experimental unit within the studies. The rat was placed in the left lateral prone position, and a 40 mm longitudinal incision was made over the lateral aspect of the right thigh. The muscles were separated to expose the lateral aspect of the femoral bone. The LBD measuring 5 mm was created using a precise motor-driven drilling machine and the femur was inserted by a 1.25 mm diameter K-wire. The scaffold or PMMA cement cylinder, with a 1 mm diameter groove in the middle, was implanted in the defect. 6 weeks after the first operation, the scaffold or PMMA was removed, autologous corticocancellous bone grafts, collected from caudal vertebrae, were weighted and grafted in the defect area. The wound and skin were closed by 4 − 0 silk sutures. 55 male SD rats weighed (mean ± SD) 152 ± 11 g were used in this study. The rats could be divided into four groups depending on the spacer filled in the defect area. Group 1 was “defect only” group (*n* = 10), without spacers in the defect area; Group 2 was “PMMA” group (*n* = 15) with PMMA in the defect area; Group 3 was “PRP-nHA/PA66” group (*n* = 15) with PRP-nHA/PA66 in the defect area, and group 4 was “PRP-FG-nHA/PA66” group (*n* = 15) with PRP-FG-nHA/PA66 scaffold in the defect area. 15 rats had to be excluded because of postoperative complications, including incision infection (*n* = 4), incision rupture (*n* = 3), scaffold and PMMA dislocation (*n* = 4), and unexplained death (*n* = 4). The 15 excluded rats belong to defect only group (*n* = 3), nHA/PA66 group (*n* = 4), PRP-nHA/PA66 group (*n* = 5) and PRP-FG-nHA/PA66 group (*n* = 3).

In group 2–4, 5 rats were randomly selected and humanely euthanized at 6 weeks after the first operation. The remaining rats were received the secondary operation. After 6 weeks, 5 rats were randomly selected and humanely euthanized. In defect only group, 5 rats were randomly selected and humanely euthanized at 12 weeks after the first operation. Random numbers were generated using the standard = RAND () function in Microsoft Excel. The group allocation was blinded to the experimenters during the conduct of the experiment, the outcome assessment and the data analysis.

### Histologic assessment

Induced membrane samples at 6 week after first-stage surgery and bone samples at 6 week after second-stage surgery were collected. Samples containing bone tissue were additionally demineralized in a 10% EDTA solution. All samples were fixed in 4% (v/v) neutral paraformaldehyde for 24 h after collection. Following dehydrated and embedded in paraffin, the tissues were cut into 5 μm sagittal sections. Then, the slides were stained with hematoxylin and eosin and bone tissue slides were additionally stained with masson’s staining. The protocols were the same as our previous study [21]. The figures were examined and captured by another group of experienced histology researchers in a blinded manner using a microscope (Olympus, Japan).

### Micro-CT measurement

6 weeks after second-stage surgery, the bone samples were scanned using a micro-CT scanner (Skyscan 1076, Belgium) at 30 μm resolution as previously described [17]. A constant volume of interest centered over the defect site, with 150 slices thick (approximately 5 mm), was selected for analysis. The two dimensional images were reconstructed. The bone volume (BV, mm^3^), bone volume density (BV/TV, %), and bone mineral density (g/cm^3^) were calculated. The callus formation was scored on a three point system as described by DeBaun MR et al. [22], where a score of 0 = pseudarthrosis (almost no callus formed), 1 = no bridging (presence of callus but no cortex bridging), 2 = incomplete bridging (one to three cortices bridged by callus), 3 = complete bridging (four cortices bridged by callus). Scoring was carried out by blinded observers.

### Statistical analysis

Statistical analysis was performed by using GraphPad Prism 9 software. All data were expressed as mean ± SD. The data between two groups were compared using independent-samples t-test. The data between three or more groups were compared by the one-way ANOVA and Tukey test. *P* < 0.05 was considered to be statistically significant.

## Results

### The structure of PRP-FG-nHA/PA66 scaffold and sustained release of VEGFA

**Fig. 1:**
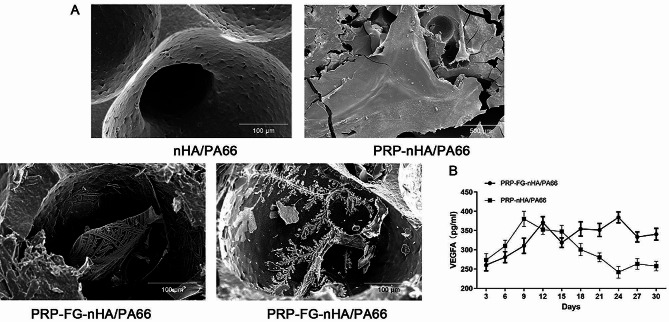
**A**, the morphology of nHA/PA66, PRP-nHA/PA66 and PRP-FG-nHA/PA66. After the addition of fibrinogen and lyophilization, the PRP-gel had a crystalline shape and attached to the walls of the pores. **B**, ELISA assay for concentration of VEGFA after 30 days of preparation. Data in mean ± SD, *n* = 5

The structure of PRP-FG-nHA/PA66 scaffold were observed by SEM. The SEM showed that the nHA/PA66 scaffold had a rough surface and the average pore diameter of nHA-PA66 scaffolds was 300–500 μm. Large pores were connected by small pores. Without fibrinogen and lyophilization, the PRP-gel could only cover the surface of the scaffold. After the addition of fibrinogen and lyophilization, the PRP-gel had a crystalline shape. The PRP-gel could permeate into the pores and attach to the walls of the pores (Fig. 1A). The average porosity of the PRP-FG-nHA/PA66 scaffold was 58.7%. The results of ELISA showed that the PRP-FG-nHA/PA66 scaffold, prepared by fibrinogen and lyophilization, could still maintain a high concentration of VEGFA after 30 days of preparation (Fig. 1B).

### PRP-FG-nHA/PA66 scaffold significantly improves angiogenesis of induced membrane

**Fig. 2:**
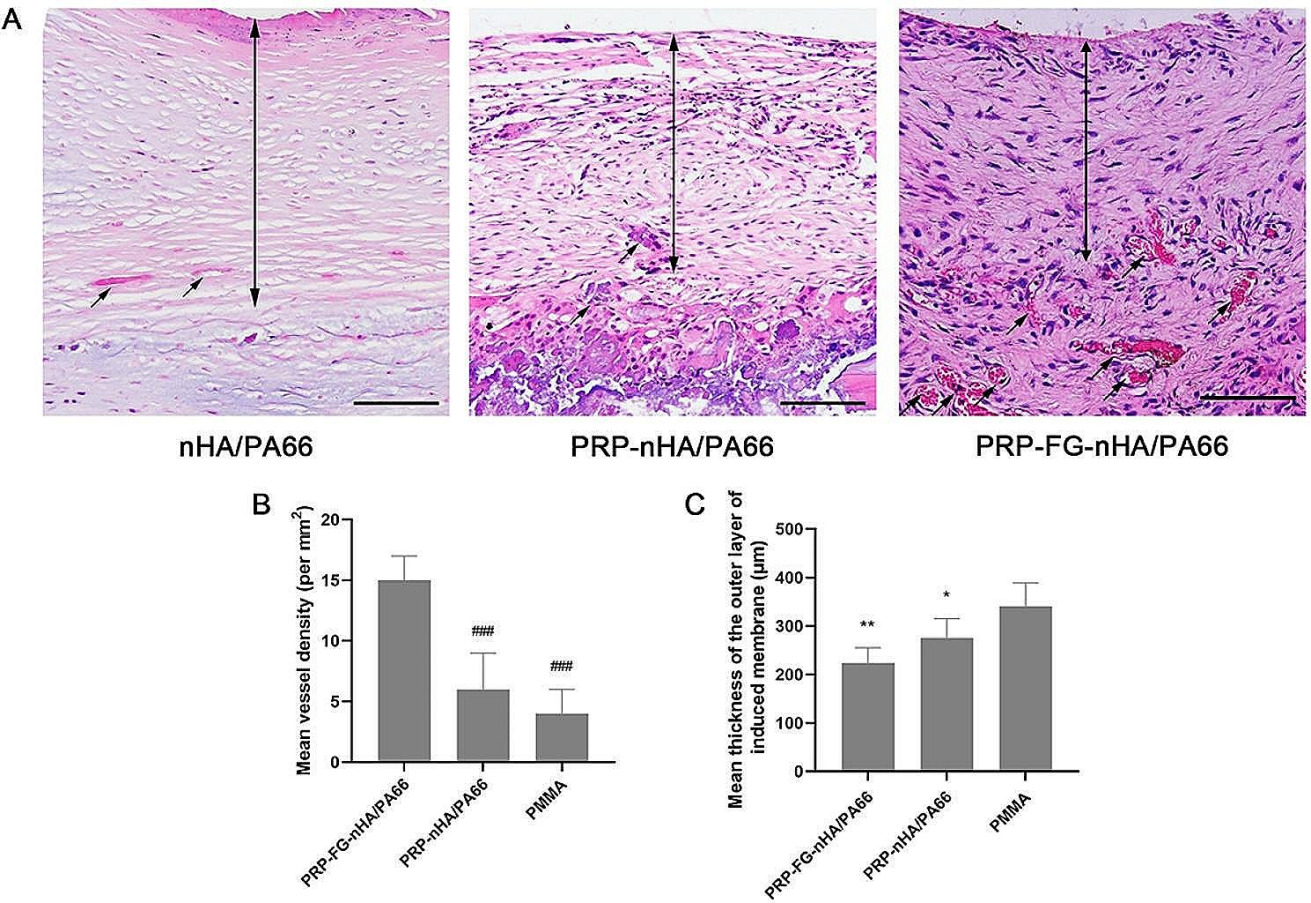
**A**, HE staining results of the induced membrane formed in the defect area after 6 weeks of first-stage operation (scale bar: 100 μm, black arrows indicate microvessels). **B** Quantitative results for the thickness of outer layers of the induced membrane. **C** Quantitative results of vessel density in induced membrane. *n* = 5, mean ± SD, ^*^p < 0.05, ^**^p < 0.01, versus PMMA group, ^###^*p* < 0.001, versus PRP-FG-nHA/PA66 group

After 6 weeks of first-stage surgery, the induced membrane was stained with hematoxylin and eosin. The results showed that the induced membrane could be roughly divided into inner and outer layers, which had different morphological features. The outer layers of induced membrane were mainly composed of fibrous connective tissue and microvessels mainly existed in inner layers. In PMMA group, the outer layer of induced membrane was mainly composed of loose connective tissue, and the inner layer was more compact than the outer layer. Although the thickness of outer layer of the induced membrane in PMMA group was greater than that of PRP-FG-nHA/PA66 group, more microvessels could be seen in the inner layer of PRP-FG-nHA/PA66 group (Fig. 2A). The histomorphometric results were consistent with the observation. According to the results, the outer layer of the induced membrane in PMMA group was the thickest, but the vessel density of the inner layer in PRP-FG-nHA/PA66 group was the highest among the three groups (Fig. 2B-C).

### PRP-FG-nHA/PA66 scaffold significantly reduced the volume of bone grafted planted in second-stage surgery

**Fig. 3:**
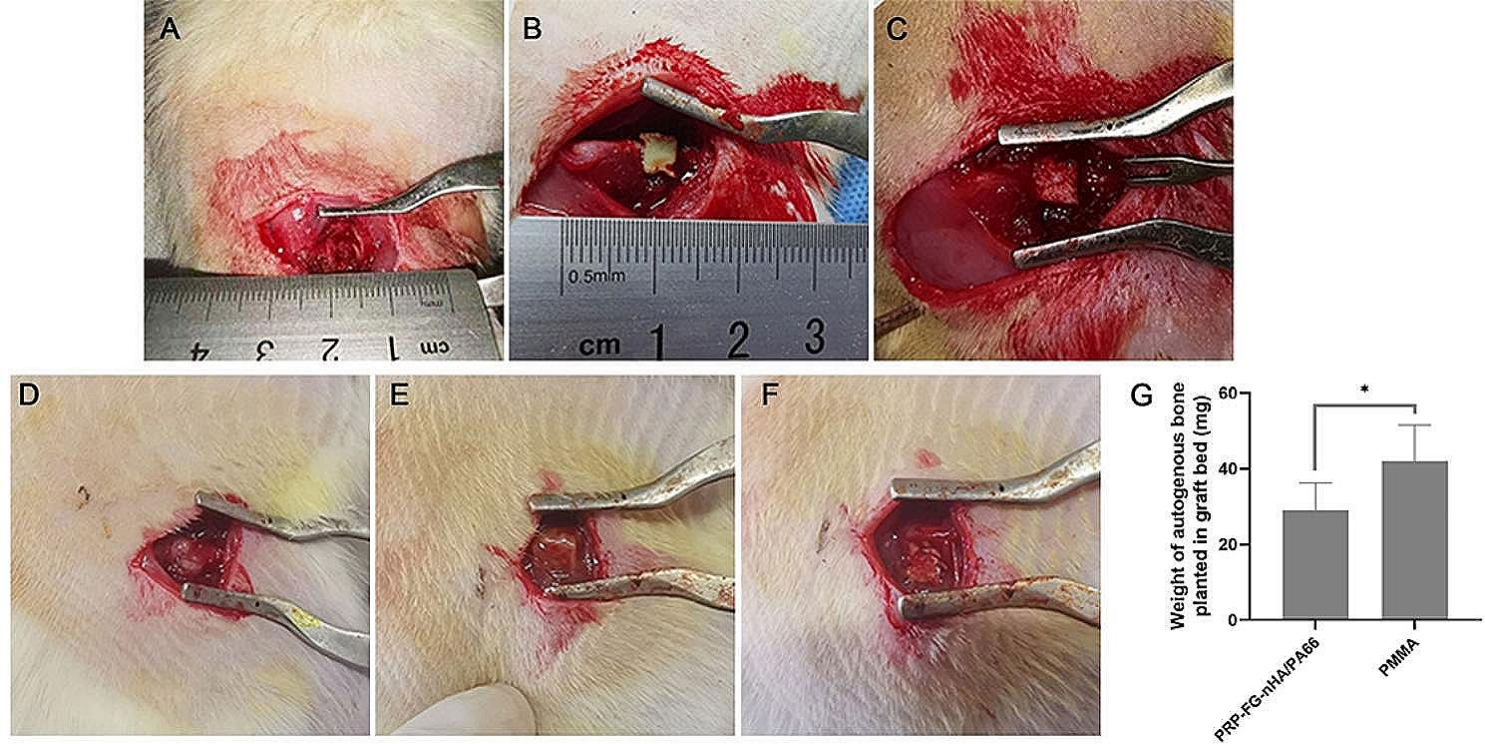
**A - F** illustrated the procedure of the establishment of induced membrane in femoral defect site. **A** Bone defect site of 5 mm in length in the right femur of the rats was prepared in the first-stage surgery. (B) PMMA and (C) PRP-FG-nHA/PA66 were prepared into 5 × 5 × 5 mm^3^ small pieces and implanted in the defect site. The induced membrane formed by (D) PMMA and (E) PRP-FG-nHA/PA66 after 6 weeks of first-stage surgery. **F** The autogenous bone was planted in graft bed in PRP-FG-nHA/PA66 group. **G** The weight of autogenous bone planted in graft bed after the removement of PMMA and PRP-FG-nHA/PA66, *n* = 5, mean ± SD, ^*^p < 0.05

Figure 3A - F illustrated the procedure of the establishment of induced membrane in the rat femoral bone defect site. Both PRP-FG-nHA/PA66 scaffold and PMMA were prepared into 5 × 5 × 5 mm^3^ small pieces with a 1 mm diameter groove in the middle before implantation. After 6 weeks of first-stage surgery, it was found that the membrane-like tissue generated around the PMMA spacer was thicker than that of PRP-FG-nHA/PA66. However, a complete induced membrane rich in blood vessels could be observed. After the removement of the spacers, the autogenous bone planted in the chamber was weighed. The results showed that the weight of autogenous bone in PRP-FG-nHA/PA66 scaffold group was significantly smaller than that of PMMA group (Fig. 3G).

### The induced membrane of PRP-FG-nHA/PA66 scaffold has higher efficacy in bone defect treatment than that of PMMA

**Fig. 4:**
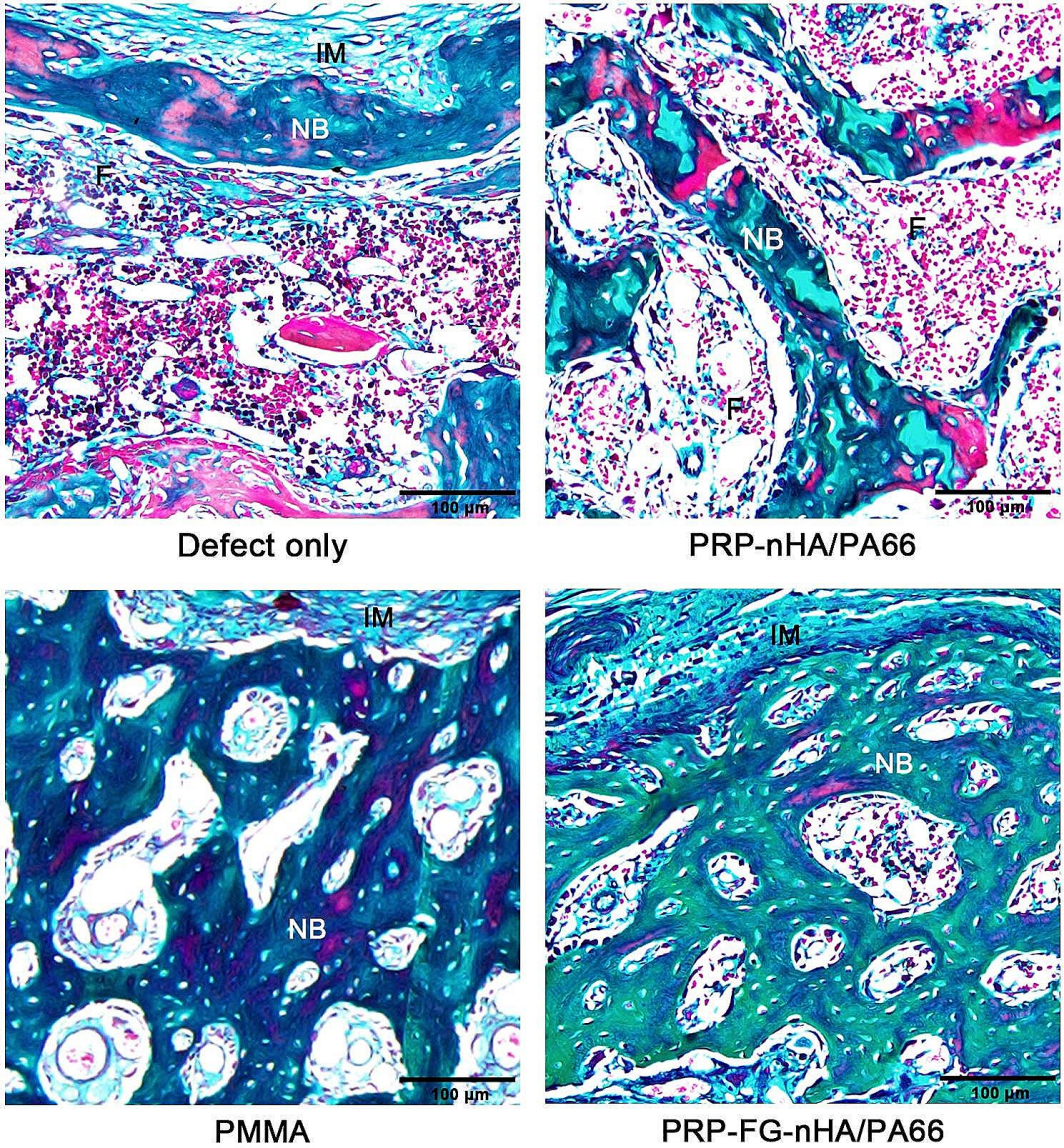
Representative images of masson’s staining from different groups. F: fibrous tissue, NB: new bone, IM: induced membrane

After 6 weeks of second-stage surgery, we performed masson’s staining and micro-CT to detect the bone formation among groups. The results of masson’s staining showed that in the defect only group, a small amount of new bone tissue was formed in the “membrane” of the bone defect area, the defect was mostly filled with fibrous connective tissue. In PRP-nHA/PA66 group, we could observe bone formation in the bone defect area, but the newly formed bones were filled with fibrous tissue. In the PRP-FG-nHA/PA66 group and PMMA group, we could observe more bone formation compared with that in PRP-nHA/PA66 group, and the bone defect was almost morphologically repaired (Fig. 4).

**Fig. 5:**
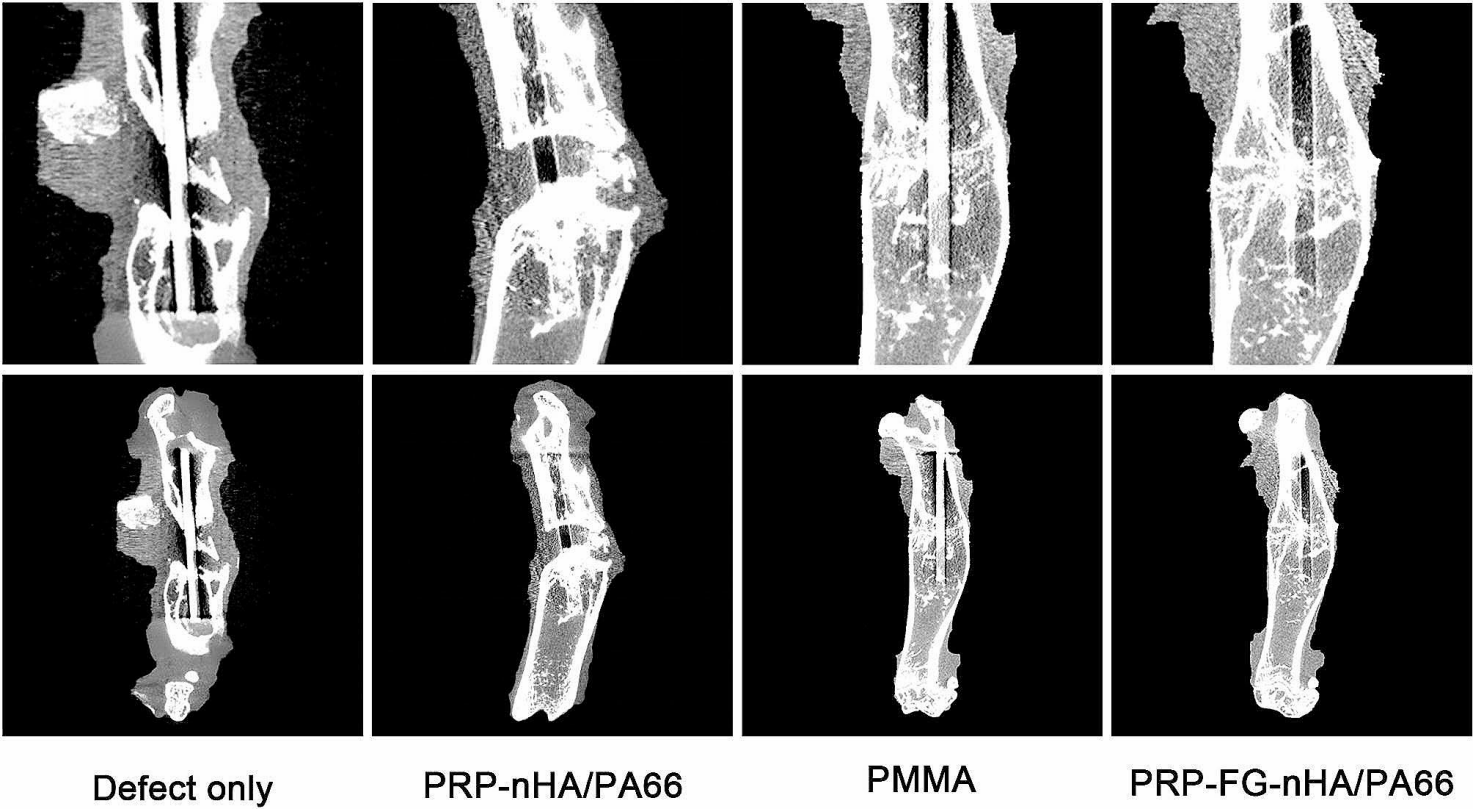
Representative images of micro CT from different groups. The top images show high magnifications of the defect areas, and the bottom images show low magnification overview of the whole femurs after 6 weeks of second-stage surgery (The straight line in the femoral marrow cavity is the K-wire)

**Fig. 6:**
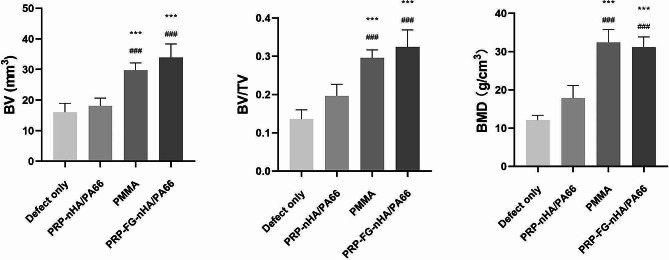
Bone volume (BV, mm^3^), bone volume density (BV/TV, %) and bone mineral density (g/cm^3^) of the three groups, *n* = 5, mean ± SD, ^***^p < 0.001, versus defect only group; ^###^*p* < 0.001, versus PRP-nHA/PA66 group

Micro CT results showed that in the defect only group, there was enveloping callus around the bone defect area. Although the area of bone defect was reduced, the bone defect was still existed. In PRP-nHA/PA66 group, little callus was observed in the bone defect area. In PRP-FG-nHA/PA66 group and PMMA group, continuous callus was observed in the bone defect area (Fig. 5). Quantitative parameters including bone volume (BV, mm^3^), bone volume density (BV/TV, %) and bone mineral density (g/cm^3^) for bone formation were obtained and statistically analyzed, the results were illustrated in Fig. 6. The PRP-FG-nHA/PA66 group and PMMA group showed significantly greater mineralized callus formation compared with PRP-nHA/PA66 group and defect only group, but no significant difference was found between the two groups in terms of the bone volume, bone volume density and bone mineral density.

Callus union was also scored based on the micro-CT scans. Defect only group had a mean score of 1.0 ± 0.71, with PRP-nHA/PA66 group having 1.8 ± 0.84, PRP-FG-nHA/PA66 group having 2.4 ± 1.14 and PMMA group having 2.8 ± 1.30. No significant difference was found between PRP-FG-nHA/PA66 group and PMMA group. Bridging of the defect, which was demonstrated by a score of two or greater, was observed in 1/5 (20%) of defect only group, 2/5 (40%) of PRP-nHA/PA66 group, 4/5 (80%) of PRP-FG-nHA/PA66 group, and 4/5 (80%) of PMMA group.

## Discussion

In 1986, Masquelet accidentally discovered the induced membrane technology. At that time, the PMMA was used only as a spacer to fill the bone defect, and the induced membrane surrounding the PMMA was retained to avoid excessive bleeding. However, subsequent studies found that this membrane induced by PMMA had many benefits, the membrane not only promoted vascularization and corticalization of the graft but also prevented its resorption [23]. In 2010, Masquelet proposed the concept of induced membrane technology, and this technology opened up new prospects for the treatment of bone defects [24].

Masquelet induced membrane technology involves two stages of surgery. Radical debridement is critical in the first surgery, especially for cases such as infectious bone defects and osteomyelitis. Debridement can remove bacterial biofilms, necrotic tissue, scars, granulation tissue, and non-vital tissues present in the bone defect area. Study has shown that conservative debridement [25] is a major factor contributing to high recurrence rate of infection. Some studies even consider infectious bone diseases as malignant conditions requiring surgical debridement [26]. Because of the radical debridement in the first surgery, large bone defects are often formed in the second stage. The defect area may even exceed two-thirds of the bone length [27]. Although these defects can be repaired in the second stage, it requires massive bone grafting to fill the chamber. The main source of autograft is anterior or posterior iliac crest, and a large amount of cancellous autogenous iliac grafting may easily cause donor site diseases, including movement limitations, perceptual disturbances, deformity and pains [28, 29]. Therefore, massive autogenous bone graft is the major disadvantage of the Masquelet induced membrane technique [30]. In our study, we used PRP-FG-nHA/PA66 instead of PMMA as an spacer in the first stage, and then we observed the morphology of the induced membrane and weighed the amount of autologous bone required for the second stage of surgery. Our results showed that the PRP-FG-nHA/PA66, as a spacer, could not only significantly reduce the amount of autogenous bone graft in the second stage, but also repair the bone defect as PMMA. Our study indicated the induced membrane of PRP-FG-nHA/PA66 scaffold had higher efficacy in bone defect treatment than that of PMMA.

Schmitz JP et al. have found that if the defect length reaches one-tenth of the bone length, the defect will exceeded the critical size for self-repair [31]. The average femoral length of SD rats in our study was about 4 cm, therefore, we established a 5 mm large segmental bone defect in the right femurs of SD rat. In our previous study, we confirmed the effective of HUMSCs-PRP gel/nHA-PA66 scaffold to promote bone regeneration in the rat LBD repair [17]. However, the previous study also has many deficiencies. First, we found the immersion of PRP gel could only enable the PRP to cover the surface of the scaffold; Second, the HUMSCs are difficult to obtain and very expensive. Because of these deficiencies, we designed a new scaffold. We used lyophilization method to make the PRP gel fully attached to the surface of the scaffold and applied it to Masquelet induced membrane technology instead of PMMA as a spacer. Our results showed the PRP-FG-nHA/PA66 scaffold had many advantages. First, the PRP-FG-nHA/PA66 was prepared with a 1 mm diameter groove in the middle before implantation. This structure can make the spacers embedded in the defect area and reduce the damage to the induced membrane during the second operation to remove the spacer; Second, according to the literature, the porosity of human cancellous bone is 40–95% [31–33]. The average porosity of the PRP-FG-nHA/PA66 scaffold in our study was 58.7%, which was similar to normal cancellous bone, which met the basic requirements of scaffolds for large bone defects; Third, because of the application of fibrin as a “glue”, PRP gel could be attached not only to the outer surface, but also to the inner micropores surface of the nHA/PA66 scaffold. Our results indicated the freeze-drying step could strengthened the attachment of PRP gel. Finally, due to the thoroughly permeation and stable PRP attachment, the PRP-FG-nHA/PA66 scaffold achieved a longer growth factor release time than the PRP-nHA/PA66 scaffold, which was beneficial for the vascularization of the induced membrane. We could observed the induced membrane was rich in microvessels after 6 weeks of PRP-FG-nHA/PA66 scaffold implantation.

In Masquelet induced membrane technology, the neovascularization of the induced membrane is very important. Studies have shown that this membrane-like tissue is rich in various angiogenic factors, such as vascular endothelial growth factor A (VEGFA), transforming growth factor-β1 (TGF-β1), and so on [20]. The induced membrane has good angiogenesis ability, so it can improve the formation of microvessels and the perfusion the local blood in the bone defect area [34]. Even in the case of some ischemic diseases (such as radiotherapy, congenital pseudarthrosis formation, etc.), the induced membranes can effectively promote angiogenesis in the bone defect area [35, 36]. However, the angiogenesis ability, an important capacity of induced membrane, gradually decreases with time [37]. Studies have shown that the neovascularization of induced membrane decreased after 6 weeks of formation, and only about 60% of microvessels remained after 12 weeks [7]. Our study showed that the induced membrane around PMMA cement, although very thick after 6 weeks of implantation, was mainly composed of fibrous tissue, only a few microvessels existed in the inner layer. Insufficient angiogenesis capacity may not only prolong the treatment time, but even lead to early dissolution of the implanted autologous bone [38].

Although the angiogenic ability of induced membrane is critical for the treatment of Masquelet induced membrane technology, PMMA cement, as a traditional membrane induction medium, has many deficiencies. PMMA has little angiogenic ability and will release heat in the procession of solidification, which is easy to cause thermal damage to surrounding tissues [6]. In recent years, many studies have used biological scaffolds instead of PMMA bone cement as membrane induction media [].As an ideal scaffold material for bone defect filling, nano-hydroxyapatite/polyamide 66 (nHA/PA66) has excellent osteoconductive and osteoinductive activity. The scaffold has been widely used in a variety of clinical surgical procedures [40]. Because nHA/PA66 scaffold has many advantages compared to PMMA, we used nHA/PA66 as the scaffold of the composite material to fill the defect area.

Previous study has shown that although many cytokines released after PRP activation are involved in the angiogenesis process, VEGFA has a dominant role among these cytokines [41]. The cytokines released after PRP activation, such as PDGF and TGF- β1, not only synergize with VEGFA, but also upregulate the expression of VEGFA and its receptors. Therefore, although many cytokines can be released after PRP activation, VEGFA is a key regulator of angiogenesis promoted by PRP. In this study, we used lyophilization method to combine PRP, FG with nHA/PA66 scaffold and analyzed the release of VEGFA. The result of ELISA showed that the PRP-FG-nHA/PA66 scaffold could release VEGFA for up to 30 days. PRP related cytokines make PRP-FG-nHA/PA66 scaffold has a good angiogenic ability.

To further demonstrate the angiogenic ability of PRP-FG-nHA/PA66 scaffold, we observed the microvessels formation of the induced membrane. At 6 weeks after implantation, neovascularization could be observed around both PMMA and PRP-FG-nHA/PA66 scaffold in the inner layer of the induced membrane. However, the vessel density around PRP-FG-nHA/PA66 scaffold was significantly greater than that in PMMA. The results of the histological observations are also consistent with what we observed in animal surgery. The results indicated that PRP-FG-nHA/PA66 scaffold we constructed had a good angiogenic ability. The high density of microvessels also indicated that the induced membrane formed by PRP-FG-nHA/PA66 scaffold had good capacity for the treatment of bone defects. These results might explain why PRP-FG-nHA/PA66, as a spacer, could significantly reduce the amount of bone graft in the second stage. There are also some limitations in our study. First, this study did not investigate the mechanism of PRP promoting the angiogenesis of induced membrane. Second, the PRP-related cytokines were not clarified. In the following study, we will optimize the preparation method of PRP and further investigate PRP-related cytokines. We hope the PRP-FG-nHA/PA66 scaffold can achieve better bone defect repair effect in future.

In conclusion, our study modified the Masquelet induced membrane technique for the repair of LBD, we used PRP-FG-nHA/PA66 scaffold instead of PMMA in the first-stage surgery. The study showed that PRP-FG-nHA/PA66 scaffold can significantly reduce the amount of second-stage cancellous autologous bone graft, and less autologous bone graft can achieve similar bone defect repair effect as PMMA. Our findings provide some reference and theoretical support for the treatment of large segmental bone defects in humans.

## Data Availability

The datasets generated during and analysed during the current study are not publicly available due to internal data storage restriction but are available from the corresponding author on reasonable request.

## References

[1] Wang J, Yin Q, Gu S, Wu Y, Rui Y (2019). Induced membrane technique in the treatment of infectious bone defect: a clinical analysis. Orthop Traumatol Surg Res.

[2] Filipowska J, Tomaszewski KA, Niedźwiedzki Ł, Walocha JA, Niedźwiedzki T (2017). The role of vasculature in bone development, regeneration and proper systemic functioning. Angiogenesis.

[3] Lee EJ, Jain M, Alimperti S (2021). Bone microvasculature: stimulus for tissue function and regeneration. Tissue Eng Part B Rev.

[4] Masquelet A, Kanakaris NK, Obert L, Stafford P, Giannoudis PV (2019). Bone repair using the Masquelet technique. J Bone Joint Surg Am.

[5] Govaert GA, Ijpma FF, McNally, Gannamani S, Rachakonda KR, Tellakula Y, Takkalapally H, Maryada VR, Gurava Reddy AV (2023). Combining non-vascularized fibula and cancellous graft in the masquelet technique: a promising approach to distal femur compound fracture management with large defects. Injury.

[6] Masquelet AC (2017). Induced membrane technique: pearls and pitfalls. J Orthop Trauma.

[7] Aho OM, Lehenkari P, Ristiniemi J, Lehtonen S, Risteli J, Leskelä HV (2013). The mechanism of action of induced membranes in bone repair. J Bone Joint Surg Am.

[8] Everts P, Onishi K, Jayaram P, Lana JF, Mautner K (2020). Platelet-Rich plasma: New Performance understandings and therapeutic considerations in 2020. Int J Mol Sci.

[9] Zhu L, Li P, Qin Y, Xiao B, Li J, Xu W, Yu B (2024). Platelet-rich plasma in orthopedics: bridging innovation and clinical applications for bone repair. J Orthop Surg (Hong Kong).

[10] Cecerska-Heryć E, Goszka M, Serwin N, Roszak M, Grygorcewicz B, Heryć R, Dołęgowska B (2022). Applications of the regenerative capacity of platelets in modern medicine. Cytokine Growth Factor Rev.

[11] Etulain J (2018). Platelets in wound healing and regenerative medicine. Platelets.

[12] Van der Bijl I, Vlig M, Middelkoop E, de Korte D (2019). Allogeneic platelet-rich plasma (PRP) is superior to platelets or plasma alone in stimulating fibroblast proliferation and migration, angiogenesis, and chemotaxis as relevant processes for wound healing. Transfusion.

[13] Oryan A, Alidadi S, Moshiri A (2016). Platelet-rich plasma for bone healing and regeneration. Expert Opin Biol Ther.

[14] Han J, Gao F, Li Y, Ma J, Sun W, Shi L, Wu X, Li T. The Use of Platelet-Rich Plasma for the Treatment of Osteonecrosis of the Femoral Head: A Systematic Review. Biomed Res Int. 2020; 2020: 1–11. 10.1155/2020/2642439 PMC7081027 32219128

[15] Clark RA (2003). Fibrin glue for wound repair: facts and fancy. Thromb Haemost.

[16] Ortiz AC, Fideles SOM, Pomini KT, Reis CHB, Bueno CRS, Pereira ESBM, Rossi JO, Novais PC, Pilon JPG, Rosa Junior GM, Buchaim DV, Buchaim RL (2021). Effects of Therapy with Fibrin glue combined with mesenchymal stem cells (MSCs) on bone regeneration: a systematic review. Cells.

[17] Liu W, Huang Y, Liu D, Zeng T, Wang J, Li A, Wang D, Wang X (2022). The combination of platelet Rich plasma gel, human umbilical mesenchymal stem cells and Nanohydroxyapatite/polyamide 66 promotes angiogenesis and bone regeneration in large bone defect. Tissue Eng Regen Med.

[18] Wang H, Li Y, Zuo Y, Li J, Ma S, Cheng L (2007). Biocompatibility and osteogenesis of biomimetic nano-hydroxyapatite/polyamide composite scaffolds for bone tissue engineering. Biomaterials.

[19] Qi XN, Mou ZL, Zhang J, Zhang ZQ (2014). Preparation of chitosan/silk fibroin/hydroxyapatite porous scaffold and its characteristics in comparison to bi-component scaffolds. J Biomed Mater Res A.

[20] Henrich D, Seebach C, Nau C, Basan S, Relja B, Wilhelm K, Schaible A, Frank J, Barker J, Marzi I (2016). Establishment and characterization of the Masquelet induced membrane technique in a rat femur critical-sized defect model. J Tissue Eng Regen Med.

[21] Wang X, Ju F, Li A, Geng S, Sun J, Liu R (2016). Nell-1 gene modified mesenchymal stem cells on biomimetic porous nano-hydroxyapatite/polyamide 66 scaffolds effectively prevent nonunion in rats. J Biomaterials Tissue Eng.

[22] DeBaun MR, Stahl AM, Daoud AI, Pan CC, Bishop JA, Gardner MJ, Yang YP (2019). Preclinical induced membrane model to evaluate synthetic implants for healing critical bone defects without autograft. J Orthop Res.

[23] Masquelet AC, Fitoussi F, Begue T, Muller GP (2000). Reconstruction Des os longs par membrane Induite et autogreffe spongieuse. Ann Chir Plast Esthetique.

[24] Masquelet AC, Begue T (2010). The concept of induced membrane for reconstruction of long bone defects. Orthop Clin North Am.

[25] Lew DP, Waldvogel FA, Osteomyelitis (2004). Lancet.

[26] Sanders J, Mauffrey C (2013). Long bone osteomyelitis in adults: fundamental concepts and current techniques. Orthopedics.

[27] Yu X, Wu H, Li J, Xie Z (2017). Antibiotic cement-coated locking plate as a temporary internal fixator for femoral osteomyelitis defects. Int Orthop.

[28] Starch-Jensen T, Deluiz D, Deb S, Bruun NH, Tinoco EMB (2020). Harvesting of autogenous bone graft from the ascending mandibular ramus compared with the chin region: a systematic review and meta-analysis focusing on complications and donor site morbidity. J Oral Maxillofac Res.

[29] Yeap MC, Tu PH, Liu ZH, Hsieh PC, Liu YT, Lee CY, Lai HY, Chen CT, Huang YC, Wei KC, Wu CT, Chen CC (2019). Long-term complications of Cranioplasty using stored autologous bone graft, three-Dimensional Polymethyl Methacrylate, or Titanium Mesh after Decompressive Craniectomy: a single-center experience after 596 procedures. World Neurosurg.

[30] Han W, Shen J, Wu H, Yu S, Fu J, Xie Z (2017). Induced membrane technique: advances in the management of bone defects. Int J Surg.

[31] Schmitz JP, Hollinger JO (1986). The critical size defect as an experimental model for craniomandibulofacial nonunions. Clin Orthop Relat Res.

[32] Morgan EF, Unnikrisnan GU, Hussein AI (2018). Bone mechanical properties in healthy and diseased states. Annu Rev Biomed Eng.

[33] Chen J, Zhang D, Zhang T, Chen C, Song Y, Liu S, Su Y, Guo S (2018). Effect of the vascularized bone components on the survival of vascularized composite allografts. J Surg Res.

[34] Wang W, Zuo R, Long H, Wang Y, Zhang Y, Sun C, Luo G, Zhang Y, Li C, Zhou Y, Li J (2020). Advances in the Masquelet technique: myeloid-derived suppressor cells promote angiogenesis in PMMA-induced membranes. Acta Biomater.

[35] Matsuhashi M, Saito T, Noda T, Uehara T, Shimamura Y, Ozaki T (2021). Treatment for postoperative infection of pathological femoral fracture after radiotherapy: two case reports and review of the literature. Arch Orthop Trauma Surg.

[36] Meselhy MA, Elhammady AS, Singer MS (2020). Outcome of Induced membrane technique in treatment of failed previously operated congenital pseudarthrosis of the Tibia. Orthop Traumatol Surg Res.

[37] Tang Q, Jin H, Tong M, Zheng G, Xie Z, Tang S, Jin J, Shang P, Xu H, Shen L, Zhang Y, Liu H (2018). Inhibition of Dll4/Notch1 pathway promotes angiogenesis of Masquelet’s induced membrane in rats. Exp Mol Med.

[38] Auregan JC, Begue T (2014). Induced membrane for treatment of critical sized bone defect: a review of experimental and clinical experiences. Int Orthop.

[39] Yu YH, Lee D, Hsu YH, Chou YC, Ueng SW, Chen CK, Liu SJ (2020). A three-dimensional printed polycaprolactone Scaffold Combined with Co-axially Electrospun Vancomycin/Ceftazidime/Bone Morphological Protein-2 sheath-core nanofibers for the repair of segmental bone defects during the Masquelet Procedure. Int J Nanomed.

[40] Li Q, Gao Q, Wang L, Liu L, Yang H, Song Y (2024). Comparison of long-term Follow-Up of n-HA PA66 cage and PEEK cage of lumbar Interbody Fusion in Multi-level degenerative lumbar diseases: a stepwise propensity score matching analysis. Orthop Surg.

[41] Melincovici CS, Boşca AB, Şuşman S, Mărginean M, Mihu C, Istrate M, Moldovan IM, Roman AL, Mihu CM (2018). Vascular endothelial growth factor (VEGF) - key factor in normal and pathological angiogenesis. Rom J Morphol Embryol.

